# Cyclooxygenase-2 expression in oligodendrocytes increases sensitivity to excitotoxic death

**DOI:** 10.1186/1742-2094-7-25

**Published:** 2010-04-13

**Authors:** Noel G Carlson, Monica A Rojas, Jonathan W Redd, Philip Tang, Blair Wood, Kenneth E Hill, John W Rose

**Affiliations:** 1 Geriatric Research, Education Clinical Center (GRECC) VASLCHCS, Salt Lake City, UT, USA; 2 Neurovirology Laboratory VASLCHCS, Salt Lake City, UT, USA; 3 Department of Neurobiology & Anatomy, University of Utah, Salt Lake City, UT USA; 4 Department of Neurology, University of Utah, Salt Lake City, UT USA; 5 Center on Aging, University of Utah, Salt Lake City, UT USA; 6 Brain Institute, University of Utah, Salt Lake City, UT USA

## Abstract

**Background:**

We previously found that cyclooxygenase 2 (COX-2) was expressed in dying oligodendrocytes at the onset of demyelination in the Theiler's murine encephalomyelitis virus-induced demyelinating disease (TMEV-IDD) model of multiple sclerosis (MS) (Carlson et al. J.Neuroimmunology 2006, 149:40). This suggests that COX-2 may contribute to death of oligodendrocytes.

**Objective:**

The goal of this study was to examine whether COX-2 contributes to excitotoxic death of oligodendrocytes and potentially contributes to demyelination.

**Methods:**

The potential link between COX-2 and oligodendrocyte death was approached using histopathology of MS lesions to examine whether COX-2 was expressed in dying oligodendrocytes. COX-2 inhibitors were examined for their ability to limit demyelination in the TMEV-IDD model of MS and to limit excitotoxic death of oligodendrocytes *in vitro*. Genetic manipulation of COX-2 expression was used to determine whether COX-2 contributes to excitotoxic death of oligodendrocytes. A transgenic mouse line was generated that overexpressed COX-2 in oligodendrocytes. Oligodendrocyte cultures derived from these transgenic mice were used to examine whether increased expression of COX-2 enhanced the vulnerability of oligodendrocytes to excitotoxic death. Oligodendrocytes derived from COX-2 knockout mice were evaluated to determine if decreased COX-2 expression promotes a greater resistance to excitotoxic death.

**Results:**

COX-2 was expressed in dying oligodendrocytes in MS lesions. COX-2 inhibitors limited demyelination in the TMEV-IDD model of MS and protected oligodendrocytes against excitotoxic death *in vitro*. COX-2 expression was increased in wild-type oligodendrocytes following treatment with Kainic acid (KA). Overexpression of COX-2 in oligodendrocytes increased the sensitivity of oligodendrocytes to KA-induced excitotoxic death eight-fold compared to wild-type. Conversely, oligodendrocytes prepared from COX-2 knockout mice showed a significant decrease in sensitivity to KA induced death.

**Conclusions:**

COX-2 expression was associated with dying oligodendrocytes in MS lesions and appeared to increase excitotoxic death of oligodendrocytes in culture. An understanding of how COX-2 expression influences oligodendrocyte death leading to demyelination may have important ramifications for future treatments for MS.

## Background

Multiple sclerosis (MS) is an inflammatory demyelinating disease of the central nervous system (CNS) that frequently occurs in young adults. Loss of oligodendrocytes that maintain the myelin sheath as well as damage to axons and loss of neurons is observed with MS [1-3]. The pathogenesis of MS is mediated through autoimmune and inflammatory mechanisms [reviewed in [3,4]]. Potential mechanisms have been studied using the animal models of MS, experimental autoimmune encephalomyelitis (EAE) [5] and Theiler's murine encephalomyelitis virus-induced demyelinating disease (TMEV-IDD) [5,6]. Antagonists of glutamate receptors (GluR) of the **α**-amino-3-hydroxy-5-methyl-4-isoxazolepropionic acid (AMPA) class of GluRs have been shown to limit the severity of disease in EAE [7-9], thus indicating how glutamate-mediated excitotoxicity could contribute to demyelination.

Glutamate is well known to contribute to injury to axons and death of neurons. However, glutamate mediated excitotoxicity is not restricted to neurons. Oligodendrocytes express GluRs [10] and are susceptible to excitotoxic death [11]. As such, oligodendrocyte excitotoxic death and demyelination in MS may share similar pathways known to contribute to neuronal excitotoxicity associated with other neurological diseases. We postulated that an important link between neuroinflammation and glutamate-mediated excitotoxicity in demyelinating disease could be mediated through the inducible isoform of the enzyme cyclooxygenase (COX) called COX-2. In our model, COX-2 expression in oligodendrocytes could render these cells more susceptible to glutamate-mediated excitotoxicity.

COX catalyzes the rate-limiting step in the generation of prostanoids from arachidonic acid. A constitutive form designated COX-1 and an inducible form, COX-2 have been identified [12]. COX-2 expression is induced in neurons of the CNS by glutamate receptor agonists [13,14]. COX inhibitors termed non-steroidal anti inflammatory drugs (NSAIDs) directed against COX-2 are neuroprotective *in vitro *[13,14] and *in vivo *[15,16] following induction of excitotoxicity. Changes in COX-2 expression by genetic manipulation can alter neuronal susceptibility to excitotoxicity. Overexpression of neuronal COX-2 renders neurons more susceptible to excitotoxicity [17] and neuronal loss in aged mice [18]. Conversely, loss of COX-2 in knockout mice decreases neuronal death following excitotoxic challenge [19]. This evidence illustrates how COX-2 expression and activity can contribute to neuronal excitotoxic cell death. If an analogous role for COX-2 were present in excitotoxicity of oligodendrocytes, we would predict that expression of COX-2 in oligodendrocytes may contribute to excitotoxic death of these cells. We have shown that in MS lesions, COX-2 was expressed by inflammatory cells [20] and oligodendrocytes [21]. Recently, we have demonstrated that COX-2 was expressed in dying oligodendrocytes at the onset of demyelination in TMEV-IDD [21]. This is consistent with a role for COX-2 in death of oligodendrocytes and demyelination. In this context, we hypothesized that increased COX-2 expression in oligodendrocytes could accentuate glutamate-mediated excitotoxic death in oligodendrocytes and that decreased COX-2 expression (or inhibition of enzymatic activity) may limit excitotoxicity and demyelination. In this study we examined the potential link between COX-2 expression in oligodendrocytes and death of oligodendrocytes in MS lesions. The potential effects of COX-2 inhibitors were examined in the TMEV-IDD model of MS along with the direct effects on decreasing excitotoxic death of oligodendrocytes in culture. Finally, we addressed whether changes in oligodendrocyte expression of COX-2 by genetic manipulation can alter sensitivity of oligodendrocytes to excitotoxic death.

## Methods

### Materials

Tissue culture media and chemistry along with the Kainic acid were purchased from Sigma Chemical Company (Saint Louis, MO). Fetal bovine serum and horse serum was purchased from Hyclone (Logan, UT). All the COX-2 inhibitors (CAY 10452, NS398 and CAY 10404) were purchased from Cayman Chemical Company (Ann Arbor, MI).

### MS spinal cord plaque

Tissue for this study was obtained at autopsy from a patient with clinical definite MS (CDMS) by McDonald criteria and Poser criteria [1,2] confirmed by MRI of brain and cervical spinal cord as well as presence of cerebral spinal fluid oligoclonal bands. Multiple cervical cord lesions consistent with demyelinating lesions were observed on MRI at the time of diagnosis. The patient had an initial aggressive course of relapsing and remitting disease followed by progressive decline. After a short course of prednisone the patient did not pursue immunotherapy. The patient expired six years later and the cervical cord was resected with an autolysis time of 5 hours. The tissue was fixed in paraformaldehyde. Studies of this autopsy specimen were approved as exempt by the University of Utah IRB in accordance with DHHS federal regulation 45CFR46 [20,22].

### TMEV-IDD model

All aspects of animal handling and care were conducted with local Institutional Animal Care and Use Committee (IACUC) approval in an Association for Assessment and Accreditation of Laboratory Animal Care (AAALAC)-approved facility (The Veterans Affairs Salt Lake City Health Care System Veterinary Medical Unit). For each time point, six mice were inoculated by IC (intracerebral) injection with 2 × 10^5 ^plaque forming units (PFU) of the DA strain of TMEV. At selected times the animals were anesthetized and then perfused with phosphaste buffered saline (PBS) containing 2% paraformaldehyde. Multiple transverse sections were made through the spinal cord at the cervical, thoracic, and lumbar levels. Scoring utilized in our studies is as follows: spinal cord, midbrain, cerebellum and cerebrum were evaluated in each animal and scored for inflammation [5,6]. The scale for inflammation is: 0 = no inflammatory cells; 1 = a few inflammatory cells in the meninges; 2 = mild meningeal inflammatory cells around blood vessels; 3 = moderate perivascular cuffing with extension into the adjacent parenchymal space; and 4 = extensive perivascular cuffing and increased parenchymal inflammation. The scale for demyelination is: 0 = none; 1 = subpial demyelination (subarachnoid inflammation with just meningitis) (<10% demyelination); 2 = extension beyond the subpial region (10-25% demyelination); 3 = large regions of white matter involvement (25-50% demyelination); and 4 = extensive white matter involvement in virtually the entire quadrant (>50% demylination). For statistical purposes multiple sections of the CNS were obtained. For example, 10 sections of the spinal cord were obtained and each quadrant of the cord section was scored providing 40 data points/mouse. Data from each group was analyzed using InStat3, a statistical software package (graph pad Prism, San Diego CA). Kruskal-Wallis Test (Nonparametric ANOVA) was used for comparisons between groups.

### Immunofluorescent confocal microscopy

Immunoreactivity was assessed with primary antibodies to mouse antigens that included anti-COX-2, (Cayman Chemicals, Ann Arbor, MI), anti-activated caspase 3 (Cell Signaling Technology, Beverly, MA) and anti-CNPase (Sigma, St. Louis, MO). Primary antibodies for human MS lesions were goat anti COX-2 (Abcam, Cambridge, MA), mouse anti-2',3'-cyclic-nucleotide 3'-phosphodiesterase (CNPase) (Sigma) and rabbit anti-activated caspase 3 (Sigma). Primary antibodies were used at dilutions established by our previous studies. Secondary fluorochrome antibodies for mouse were donkey FITC conjugated anti-rabbit and Cy5 conjugated anti-mouse/rat (Jackson immunoresearch laboratories, West Grove, PA) and for human tissue were donkey FITC anti-goat, Cy5 anti-mouse and C3 anti-rabbit. Secondary antibodies were used at concentrations from our previous established results. The combined primary antibodies were added and incubated overnight in a humidified chamber at 4°C. Conjugated secondary antibodies were added for 1 hour at room temperature (RT). Negative protocol controls were 20 μg/ml normal mouse/rat serum and 30 μg/ml normal rabbit serum. Coverslips were mounted using ProLong Gold anti-fade mounting media (Molecular Probes Inc., Eugene, OR). Personal Confocal Microscopy PCM-2000 (NIKON, Melville, NY) utilizes Argon, green and red HeNe lasers to acquire images from the three different fluorochromes. Simple Personal Confocal Image program (PCI, Compix, Cranberry Township, and PA) was used to acquire digital images. The fluorochromes were resolved from three different image channels. The FITC label was detected with the Argon laser at 488 nm, Cy5 with the red Argon laser at 633 nm and Cy3 was visualized with the green HeNe laser at 563 nm. Tissues were individually scanned with each respective laser filter. Most images were acquired using the multi-focal program (z-focus) to create a stereopsis image. The three different images were merged together to acquire the final triple-colored image. propidium iodide (PI) image converted to blue color during merge.

### Dispersed oligodendrocyte cultures and excitotoxicity assay

Dispersed oligodendrocyte cultures were prepared from P1 mouse pups essentially as described [23]. Oligodendrocytes were plated in 96 well plates and photographed using phase contrast microscopy prior to treatment with kainic acid. The same fields were photographed 24 hours after KA treatment. For toxicity experiments, oligodendrocytes were identified by staining with olig-1 and scored as dead by staining with PI. The percent survival was calculated by dividing the number of live (cells not stained with PI) observed after KA treatment divided by the number of cells present prior to KA treatment. Three or more fields were captured for each treatment group. This assay is similar to our previous published assays to determine neuronal survival following excitotoxicity [14,24,25]. The percent survival was calculated as percent control relative to the survival observed with no KA treatment. Background death was typically less than 25%.

### Organotypic spinal cord (slice) cultures

Organotypic spinal cord slice cultures were prepared as previously described [26]. Spinal cords were rapidly removed from P10 mouse pups after the animals were sacrificed. Lumbar spinal cords were collected under sterile conditions and sectioned transversely into 350 μm thick sections using a McIlwain tissue chopper (Mickle Lab Engineering, Gomshall, Surrey U.K.). The slices were transferred in Hank Buffered Salt Solution (HBSS) (Invitrogen) and placed on the surface of a 30 mm diameter Millipore Millicell-CM porous (0.04 μm) membranes with 4-5 slices/membrane. The membranes were placed in 6-well plates in 1 ml of minimal essential media (MEM) with 25% horse serum, glutamine (2 mM) and 10 mM HEPES buffer (pH 7.4). Cultures were incubated at 37°C in a 5% CO_2_/95% humidified environment. Explants were maintained in culture for 2 days before use in experiments. Relative toxicity was calculated as the number of dead cells per area in the white matter and gray matter zones. The number of dead cells stained with activated caspase 3 in the white matter zone was assessed 20-24 hours after the addition of KA.

### Statistical analysis

Data was analyzed using InStat3, a statistical software package (graph pad Prism, San Diego CA). Assessment of significance of cell death between groups was done using ANOVA Tukey-Kramer multiple comparisons test. All comparisons satisfied the Kolmogorov and Smirnov assumption test for Gaussian distributions thus allowing parametric analyses.

### Transgenic mice

The DNA construct used to generate the transgenic mice designed to over-express COX-2 in oligodendrocytes contained a 3.9 kb promoter region from the CNPase promoter that contains the CNP1 and CNP2 promoters [27] in a pBS/SK vector (provided by Michel Gravel McGill University). An intermediate construct was generated with a 700 bp fragment cut with XhoI containing the poly A sequence and was ligated downstream from the CNP promoters following linearization with XhoI. The resulting vector was subsequently cut with HindIII and BamHI (in sites between the poly A region and the CNP promoters) and a 2 kb fragment (HindIII/BclI) containing the human COX-2 gene (hCOX-2) (provided by Steve Prescott, University of Oklahoma) was ligated into the vector. The desired clone containing the CNP promoters upstream of the hCOX-2 gene followed by a poly A sequence was confirmed by DNA sequence analyses (University of Utah sequencing core laboratory). A 6.6 Kb fragment from this clone containing the promoter regions, hCOX-2 gene and poly A region was generated following digestion with XhoI/XbaI and was purified and subsequently injected into embryos to generate the transgenic mice (performed at the University of Utah transgenic core laboratory). Positive clones were screened using PCR primer pairs specific to the hCOX-2 gene (Primer pairs: forward primer = 5'-TGT GCT TAA ACA GGA GCA TCC, reverse primer = 5'-TTG AAG TGA TAG CCA CTC AAG, yielding a 132 bp PCR product).

COX-2 knockout mice were purchased from Taconic Farms (Germantown, NY). Post-natal pups used as a source of oligodendrocytes for cultures were generated from a cross with a homozygous knockout (COX-2 -/-) male and a heterozygous knockout female (COX-2 +/-). The mouse pups were screened with the primer sets outlined [28]. The sequences of the primers are: wild type forward = 5'-ACA CCT TCA ACA TTG AAG ACC, KO target forward = 5'-ACG CGT CAC CTT AAT ATG CG, COX-2 reverse = 5'-ATC CCT TCA CTA AAT GCC CTC. PCRs with all three primers generate products of about 700 bp for wild-type and 875 bp for the knock-out.

## Results

### COX-2 expression in dying oligodendrocytes in an MS lesion

**Figure 1:**
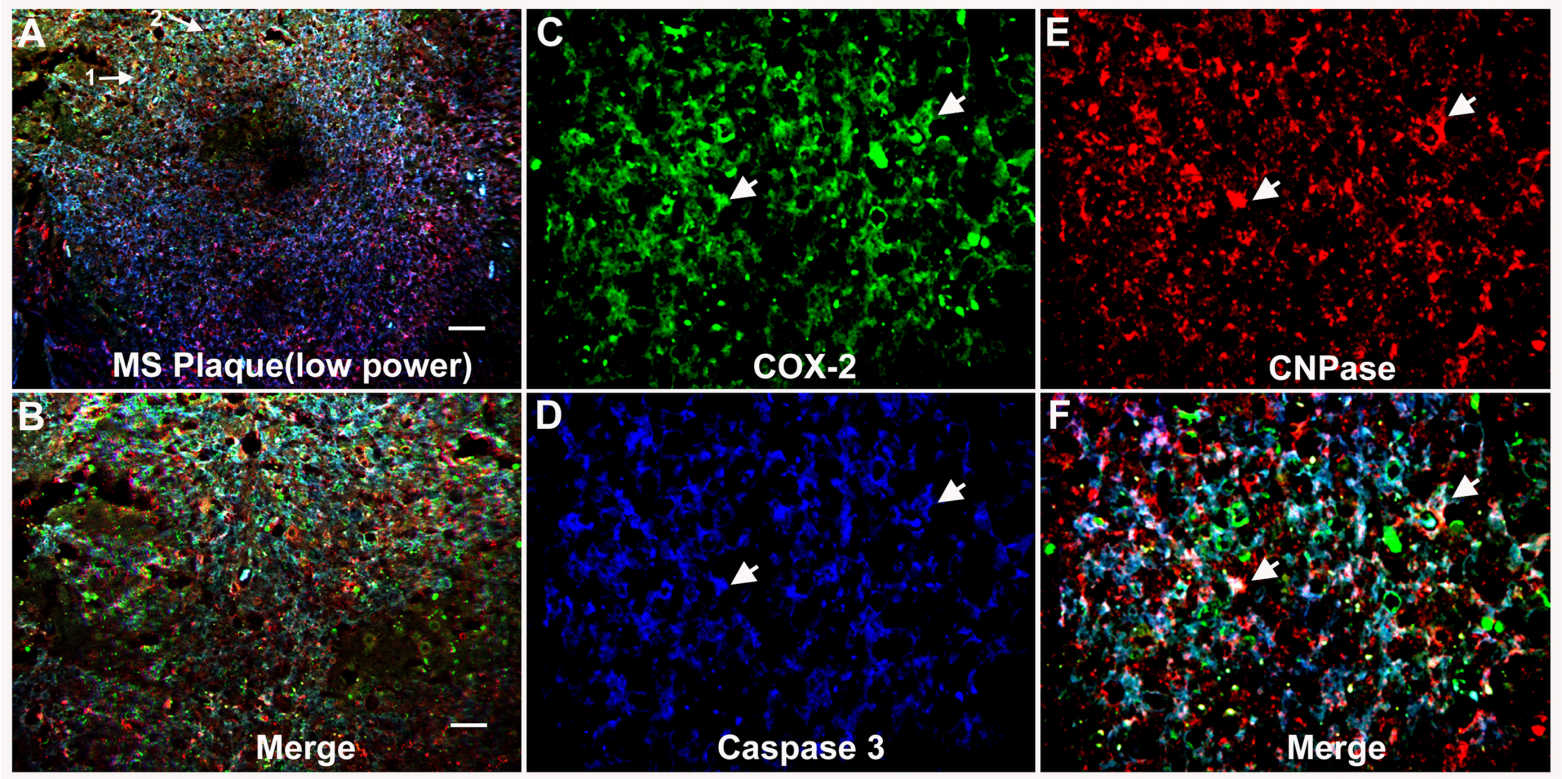
**Expression of COX-2 in dying oligodendrocytes in an MS lesion**. An MS spinal cord lesion was examined by confocal immunofluorescence after probing with antibodies to the oligodendrocyte marker CNPase (red), COX-2 (green) and activated caspase 3 (blue). (A) A low power image (10×) of the plaque shows the area of demyelination (bar = 80 μm). Two regions (see arrows 1 and 2) indicate the areas shown in higher magnification (40×) for panels B-F (bar in B = 20 μm). (B). The three color image for the region shown by arrow 1 in panel A. Note the co-expression of COX-2, activated caspase 3 and CNPase as indicated by white. (C-E) Individual channels are shown COX-2 (C), activated caspase 3 (D) and CNPase (E). The three color merged image is shown in panel F. Two examples of labeling of all three antigens (appearing white) are indicated with arrows.

We have shown previously that COX-2 is expressed in dying oligodendrocytes at the onset of demyelination in the TMEV-IDD model of MS [21]. In order to assess whether COX-2 might also be associated with dying oligodendrocytes in MS lesions, we stained MS lesions with an oligodendrocyte marker (CNPase) along with a marker for cell death (activated caspase 3) and asked whether COX-2 was associated with these markers. As seen in Figure 1, COX-2 was extensively associated with oligodendrocytes that contained activated caspase 3. This indicates that like the lesions in the TMEV-IDD model, dying oligodendrocytes in MS lesions can also express COX-2.

### The effect of COX-2 inhibitors on demyelination in TMEV-IDD

**Figure 2:**
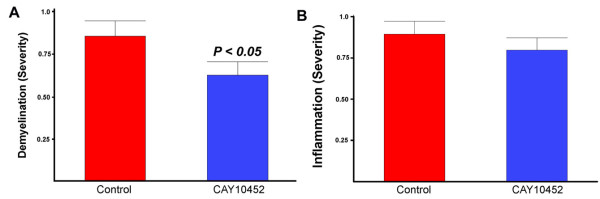
**Therapeutic effect of the COX-2 inhibitor CAY 10452 in TMEV-IDD**. **The COX-2 inhibitor CAY10452 decreases demyelination**. Spinal cord sections from control and CAY10452 (35 days after infection with TMEV) were stained with H&E and LFB and scored for inflammation and demyelination as described in methods. Comparisons between control and CAY10452 groups were done using the Mann-Whitney nonparametric t-test and the p values indicated above the error bars (SEM).

If the COX-2 expressed in oligodendrocytes in the TMEV-IDD model of MS contributes to cell death then inhibitors of this enzyme would be predicted to contribute to cell viability. In order to test this possibility, the effect of COX-2 inhibitors on demyelination was examined in the TMEV-IDD model. As seen in Figure 2, there was a significant reduction in demyelination when COX-2 inhibitors were administered two weeks after infection with TMEV. Interestingly, there was no effect of COX-2 inhibitors on the parameters of inflammation (see methods). These results are consistent with COX-2 contributing to oligodendrocyte death leading to demyelination.

### Inhibition of COX-2 protects white matter excitotoxic death in spinal cord slice cultures

**Figure 3:**
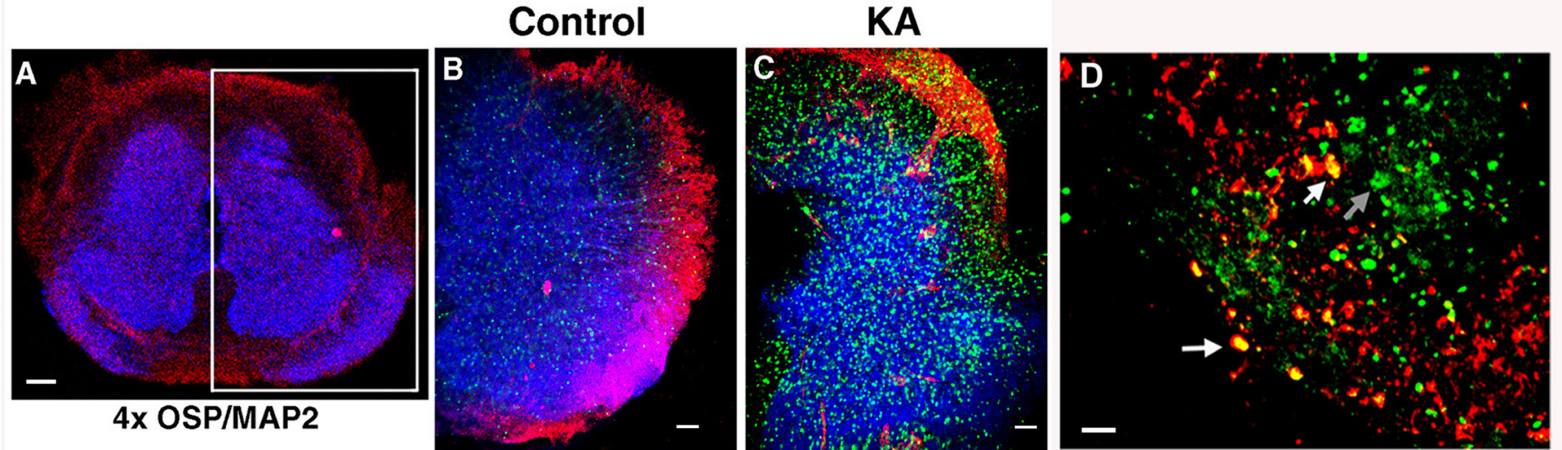
**Excitotoxicity in the spinal cord explant cultures. A) **A spinal cord culture was stained for expression of the neuronal marker MAP-2 (Blue) and for the oligodendrocyte marker oligodendrocyte-specific protein (OSP) (Red). One hemisphere is boxed to show the regions from other slices which appear in panels B and C (bar = 400 μm). Hemispheres of a slice cultures stained for MAP-2,(Blue), OSP (Red) and activated caspase 3 (green) are shown for untreated **(B) **control and kainic acid (KA) **(C)**. (Magnifications are 2× for A and 4× for B and C, bar = 80 μm for B, C and = 20 for D μm). **(D)**. A higher magnification (60×) of the white matter region showing oligodendrocytes stained for CNPase (red) and activated caspase 3 (green) and co-labeling with both as yellow. Examples of oligodendrocytes containing activated caspase 3 are shown (see white arrows). An example of a cell labeled for activated caspase 3 which is not an oligodendrocyte (and likely a motor neuron) is shown with a gray arrow.

**Figure 4:**
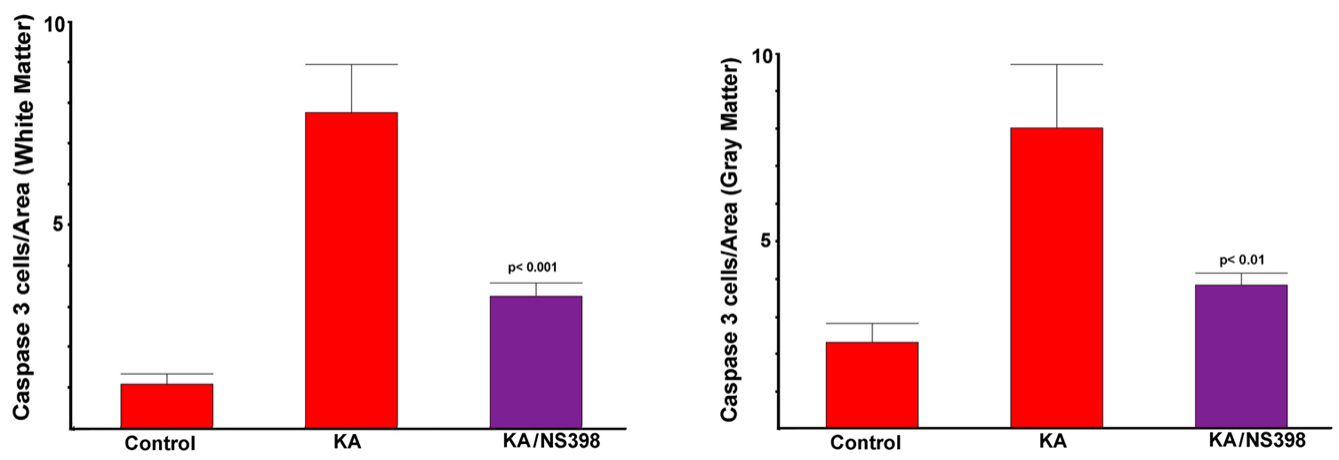
**COX-2 inhibitor mediated decrease of KA-induced activated caspase 3 in white matter and gray matter**. The appearance of cells stained with the marker for cell death (activated caspase-3) was assessed in four spinal cord sections for white matter (left) and gray matter (right) with treatments of vehicle (control), kainic acid (KA) or kainic acid with the COX-2 inhibitor NS398 (KA/NS398). Error bars are SEM and *P *values determined by ANOVA Tukey-Kramer. This is a representative experiment which has been repeated in two other experiments.

The previous findings are consistent with a role for COX-2 contributing to the loss of oligodendrocytes in demyelinating lesions. One way in which oligodendrocytes can be lost in demyelinating disease is through GluR-mediated excitotoxic death. Oligodendrocytes express GluRs [10] and are susceptible to excitotoxic death [11]. Further, inhibitors of GluRs can decrease demyelination in the EAE model of MS [7-9]. In order to test whether COX-2 inhibitors could protect white matter oligodendrocytes against excitotoxic death, an *in vitro *spinal cord slice culture system was used. This system retains neuro-anatomical relationships and allows the examination of compounds such as COX-2 inhibitors that could protect against excitotoxic death [26]. As seen in Figure 3, the GluR agonist Kainic Acid (KA) produces a robust induction of white matter cell death as indicated by the appearance of marker for cell death activated caspase 3. This marker for cell death has been observed in excitotoxic death of oligodendrocytes [29]. However, addition of the COX-2 inhibitor NS398 produced greater than a two-fold reduction in the amount of activated caspase 3 in white matter (Figure 4). COX-2 inhibitors also diminished a similar amount of KA-induced gray matter excitotoxicity. This result in gray matter is consistent with other reports showing that inhibition of COX-2 protects against neuronal excitotoxic death [13-17,26].

### GluR-induced expression of COX-2 in purified dispersed oligodendrocyte cultures

The previous results are consistent with a role for COX-2 in oligodendrocyte death. However, the previous experiments with spinal cord slice cultures do not distinguish whether the protective effects of COX-2 inhibitors are directed towards oligodendrocytes or mediated through other cell types. In order to examine the direct effects on oligodendrocytes we used a cell culture system with dispersed oligodendrocytes purified from post-natal mice (see methods). This system has two unique advantages. The first advantage is that the direct effects of COX-2 inhibitors on oligodendrocyte viability can be examined independent of other cell types. Another advantage is that these effects can also be examined for oligodendrocyte precursor cells in undifferentiated cultures. The latter is important to infer potential implications on oligodendrocyte precursor cells that contribute to remyelination.

**Figure 5:**
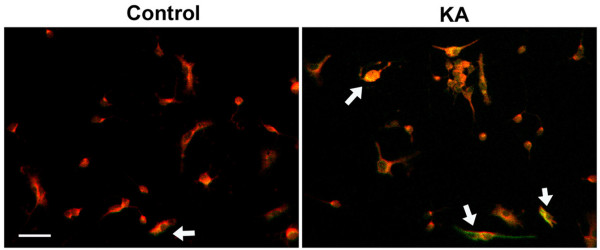
**COX-2 is induced in oligodendrocytes by Kainic Acid (KA)**. Dispersed oligodendrocyte cultures were treated with either vehicle (Control) or KA and examined 24 hours later by confocal immunofluorescence. COX-2 expression (green) is seen in the cells labeled with the oligodendrocyte specific marker Olig-1 (red), co-labeling appears yellow (see arrow). Magnification bar = 20 μm.

In neurons, activation of GluRs induces COX-2 expression [13,14] which can contribute to excitotoxic neuronal death. In order to determine whether a similar effect of GluR activation occurs for oligodendrocytes, dispersed cultures were treated with sub lethal doses of KA and the amount of COX-2 expression examined by immunofluorescent confocal microscopy. As seen in Figure 5, cultures treated with KA show a robust induction of COX-2 24 hours after KA treatment when compared to control cultures. This is consistent with a potential role of COX-2 in excitotoxic death of oligodendrocytes.

### COX-2 inhibitors protect against excitotoxic death of oligodendrocytes in dispersed cultures

**Figure 6:**
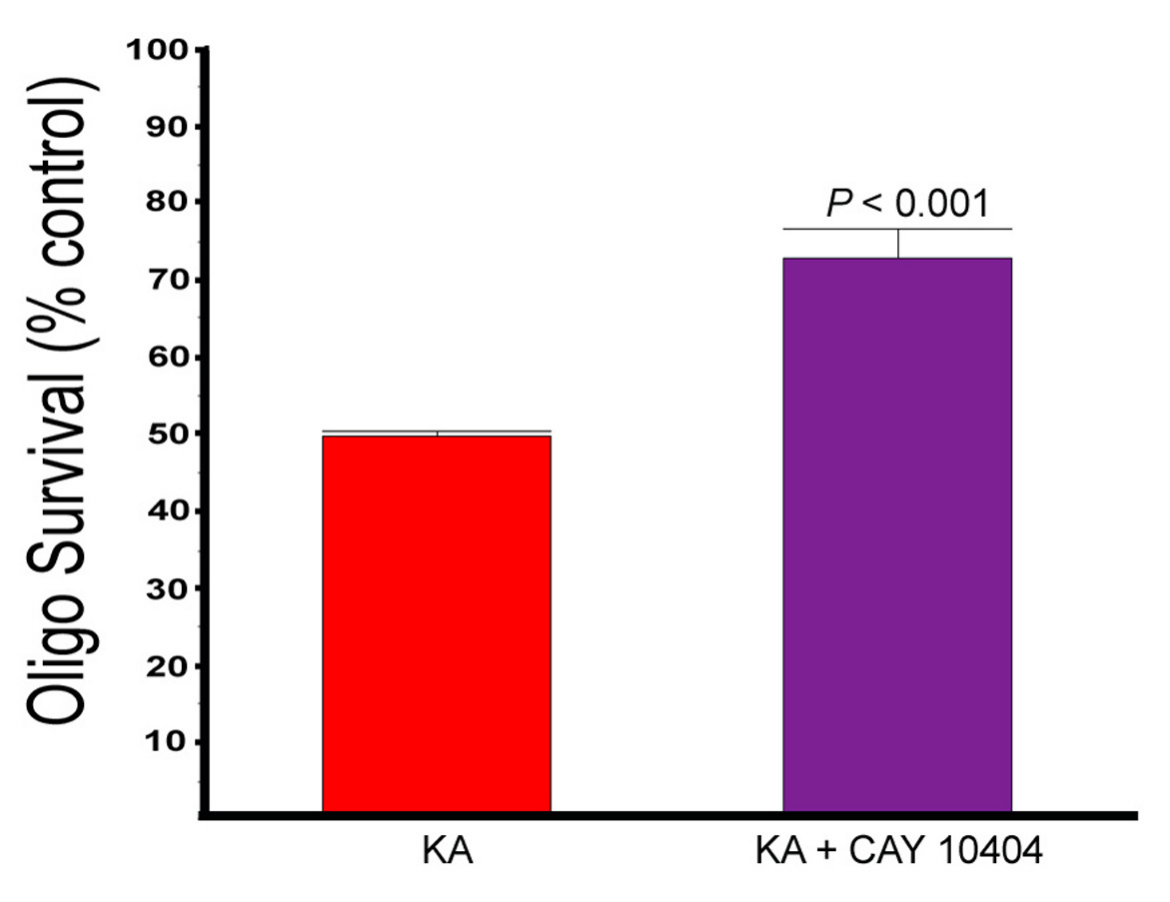
**Protection of oligodendrocytes with the COX-2 inhibitor (CAY10404)**. Dispersed oligodendrocyte cultures were treated with KA in the presence or absence of CAY 10404 (10 uM) and analyzed 24 hours later for cell death. Error bars are SEM.

The potential protective effect of the COX-2 inhibitor CAY 10404 was examined in dispersed oligodendrocytes treated with KA. As seen in Figure 6, treatment with COX-2 inhibitor resulted in a 1.5 fold increase in surviving KA-treated oligodendrocytes at 24 hours. This result indicates that COX-2 expression in oligodendrocytes increases excitotoxic death.

### Increased expression of COX-2 in oligodendrocytes enhances excitotoxic death

**Figure 7:**
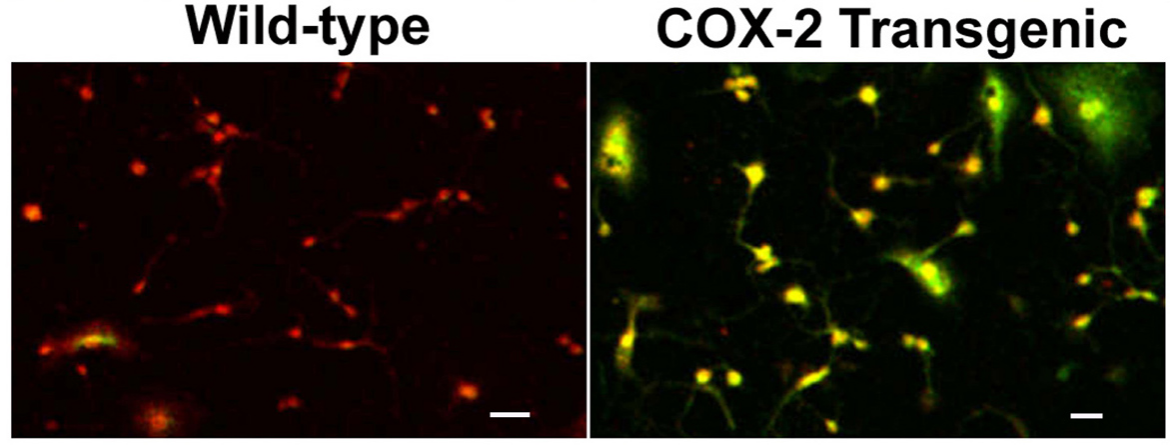
**Cultures of Oligodendrocytes derived from COX-2 transgenic mice over-express COX-2**. Dispersed wild-type cultures were prepared from wild-type and transgenic mice the over-express COX-2 specifically in oligodendrocytes (generated with the CNPase promoter fused to the human COX-2 gene. The oligodendrocyte marker to Olig-1 appears red and COX-2 appears green. Co-expression of both appears yellow. Magnification bar = 40 μm.

**Figure 8:**
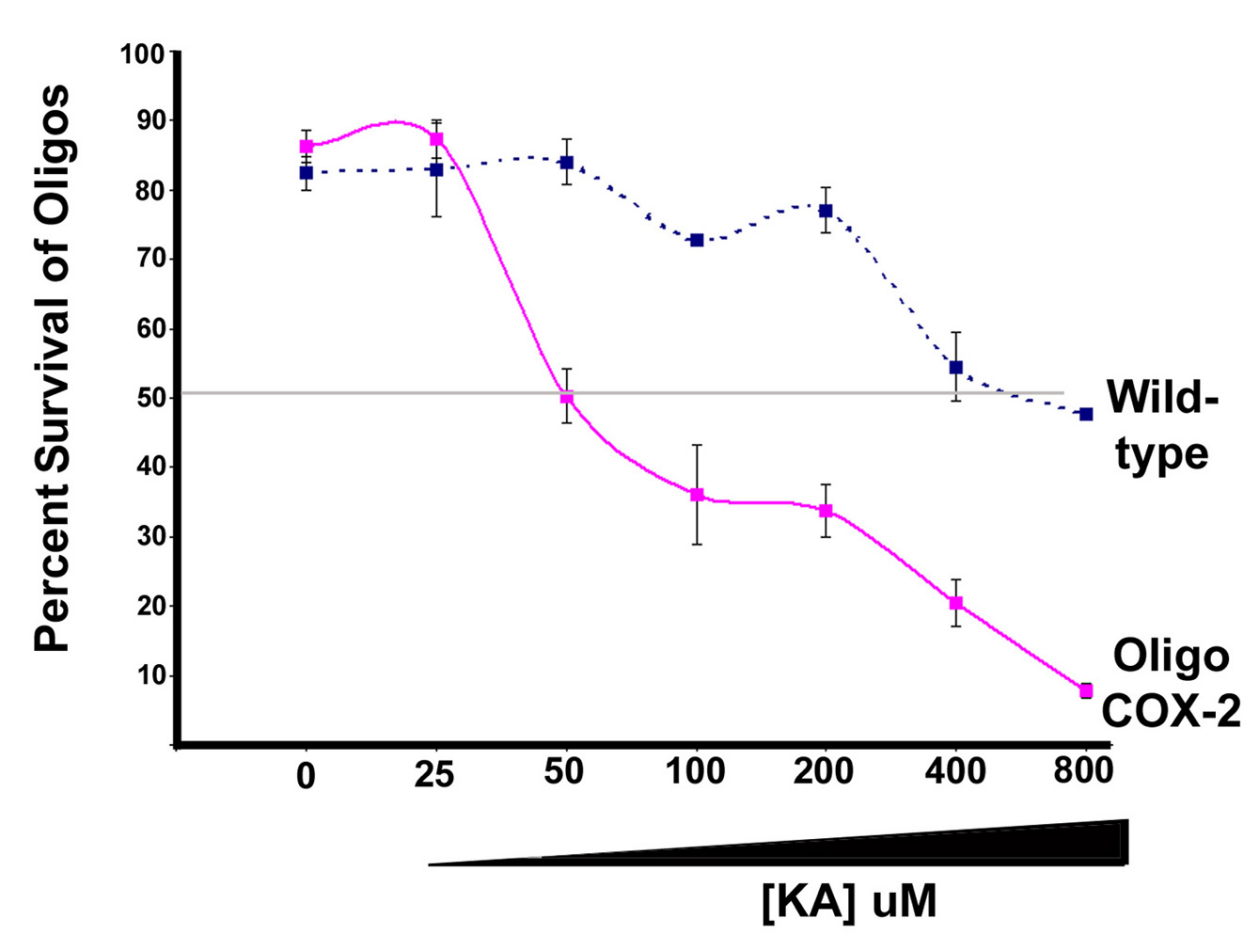
**Vulnerability of wild-type and Oligo-COX-2 transgenic oligodendrocytes to KA-induced excitotoxicity**. Dispersed oligodendrocytes were treated to varying concentrations of KA and examined for cell death 24 hours later. Oligodendrocytes derived from the transgenic animals were 8-fold more sensitive to KA than wild-type. Note, the x-axis is not a linear scale.

The previous results with COX-2 inhibitors provide supportive evidence for a role for COX-2 in excitotoxic death of oligodendrocytes. However, one potential caveat to these results is that COX-2 inhibitors may have off-target activities [30] that may promote protective effects independent of COX-2 inhibition. Therefore, we used genetic manipulation to alter COX-2 expression in order to assess whether changes in the expression have an effect on oligodendrocyte vulnerability to excitotoxic death. A transgenic mouse was generated that was designed to increase expression of COX-2 specifically in oligodendrocytes. This was achieved by linking the human COX-2 gene downstream from the oligodendrocyte promoter for the CNPase gene (see methods). The human COX-2 gene has essentially the same catalytic properties as the endogenous mouse COX-2 gene, but contains some distinct amino acid sequences that make it uniquely detectable with human COX-2 specific antibodies (see methods). When oligodendrocytes were isolated from these transgenic mice and probed with an antibody for COX-2 (that can detect both mouse and human COX-2), it was apparent that the oligodendrocytes derived from the transgenic mice exhibit a robust increase in COX-2 expression compared to wild-type oligodendrocytes (Figure 7). In order to test our hypothesis that COX-2 expression in oligodendrocytes increases sensitivity to excitotoxic death, these COX-2 transgenic oligodendrocytes were compared to wild-type oligodendrocytes for their susceptibilities to KA induced excitotoxic death. As seen in Figure 8, the KA concentration response curve for the transgenic COX-2 oligodendrocytes was shifted to the left when compared to that seen with wild-type oligodendrocytes, indicating that the transgenic COX-2 oligodendrocytes are more sensitive to KA-induced excitotoxic death. Comparison of the concentrations of KA required to kill 50% of the cells indicates that the COX-2 transgenic oligodendrocytes are eight-fold more sensitive to KA compared to wild-type.

### Loss of COX-2 expression makes oligodendrocytes less susceptible to excitotoxicity

**Figure 9:**
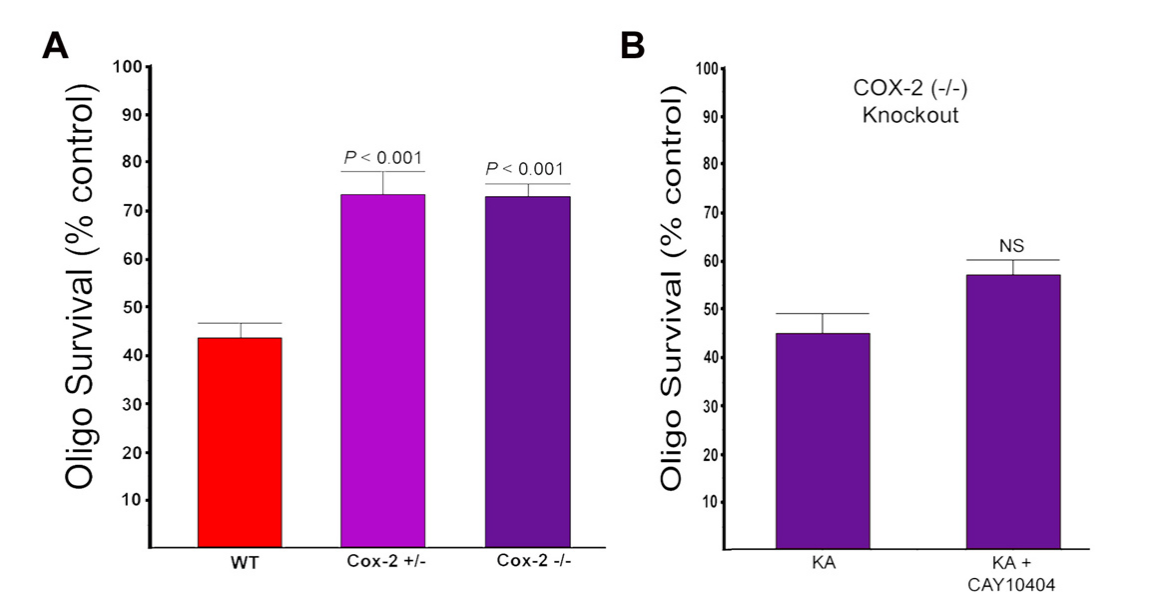
**Sensitivity of oligodendrocytes from COX-2 knockout mice to excitotoxic death and protection with COX-2 inhibitors**. (A) Oligodendrocytes from COX-2 knockout mice are more resistant to KA-induced excitotoxicity. Dispersed oligodendrocyte cultures were prepared from wild-type (WT), heterozygous COX-2 knockout (COX-2 +/-) and homozygous COX-2 knockout (COX-2 -/-) and treated with KA. Surviving cells were scored 24 hours after KA treatment. This is the average of two independent experiments. (B) The COX-2 inhibitor CAY 10404 does not protect COX-2 knockout oligodendrocytes from KA-induced death. Dispersed oligodendrocyte cultures were prepared from COX-2 -/- mice and treated with KA in the presence or absence of CAY 10404 (10 uM). Viability was assessed 24 hours after treatment with KA. There was no significant increase in the number of surviving oligodendrocytes in the CAY 10404-treated group. Error bars are SEM.

As noted earlier, a decrease in COX-2 activity after treatment with COX-2 inhibitors resulted in increased survival following an excitotoxic challenge with KA (see Figures 4 and 6). An alternative approach to decreasing COX-2 activity is to use oligodendrocytes derived from COX-2 knockout mice. As seen in Figure 9, oligodendrocytes derived form COX-2 knockout mice showed a significant (nearly two-fold) increase in survival to KA-induced excitotoxic death. Interestingly, the same degree of resistance to excitotoxic death was observed for both the homozygous COX-2 knockout oligodendrocytes (COX-2 -/-) as with the heterozygous (COX-2 -/+) oligodendrocytes. This result indicates that complete elimination of COX-2 activity is not required for maximal protection of oligodendrocytes under these conditions and that merely reducing the activity two-fold of COX-2 (by gene dosage) results in maximal protection against excitotoxic death. This specific COX-2 inhibitor (CAY 10404) also did not produce a significant increase in survival of the COX-2 (-/-) oligodendrocytes, consistent with the protective effect of this inhibitor mediated through its ability to block COX-2 activity (Figure 9).

## Discussion

In this study we demonstrated that COX-2 was expressed in dying oligodendrocytes in MS plaques in the cervical spinal cord from an MS patient. This indicates that MS lesions may share similar pathology as was seen in the TMEV-IDD model of MS where we reported that COX-2 was also expressed in dying oligodendrocytes at the onset of demyelination. These results infer that COX-2 may play a role in oligodendrocyte death and demyelination. We have extended these observations to show that COX-2 inhibitors decrease the amount of demyelination in TMEV-IDD. We have further demonstrated that COX-2 inhibitors protect oligodendrocytes in culture from excitotoxic death and that increased COX-2 expression increases excitotoxic death of oligodendrocytes while decreased COX-2 expression diminishes excitotoxic death. Combined, these results strongly support a role for COX-2 expression in oligodendrocytes as a contributing component in excitotoxic death of oligodendrocytes and a potential contributor to demyelinating disease. Our results may also have important implications for a role of COX-2 in remyelination as well. The purified oligodendrocytes in our dispersed cultures were composed of greater than 90% oligodendrocyte precursor cells as indicated by the presence of nuclear olig1 staining [31,32] (data not shown). As such, COX-2 expression contributes to loss of precursor cells and subsequently limits potential remyelination. In this context, COX-2 inhibitors may contribute to oligodendrocyte precursor cell viability and may help with remyelination in cases where precursor cells may be limited.

These findings extend our earlier observations that COX-2 is expressed in oligodendrocytes in MS lesions and that COX-2 is expressed in dying oligodendrocytes at the onset of demyelination in the TMEV-IDD model of MS [21]. These findings suggest that COX-2 inhibitors may have potential therapeutic application to MS. However, relatively little is known about how NSAIDs may limit disease in MS. There are reports of clinical use of NSAIDs for MS in management of side effects associated with IFN therapies [33] and aspirin use for limiting the severity of MS-related fatigue [34] and premenstrual associated pseudoexacerbations [35]. However, these studies were not designed to test the potential for limiting demyelination in disease and there are no other reports of therapeutic effects of NSAIDs for MS. In contrast to these limited examples of NSAID use with MS disease, COX inhibitors have been tested for their ability to limit disease in animal models of MS.

Studies with COX-2 inhibitors in animal models of MS also support a role for COX-2 as a contributor to disease pathology. Two groups have reported that administration of COX-2 inhibitors in EAE diminished the severity and incidence of disease and decreased demyelination and inflammation [36,37]. In both cases, the therapeutic effects in EAE were only observed when the COX-2 inhibitors were initiated immediately after immunization and maintained throughout the course of the study. Miyamoto and colleagues [37] also observed an improvement in EAE when the COX-2 inhibitor Celecoxib was initiated at onset of clinical symptoms (40% improvement in combined clinical score). Miyamoto et al., suggest that the therapeutic effect of Celecoxib in the induction phase of monophasic EAE is in part due to COX-2 independent actions of this drug [37]. They found that Celecoxib-induced improvements in EAE clinical scores were equivalent in wild-type and COX-2 knockout mice [37]. Another COX-2 inhibitor nimesulid, showed no therapeutic effects in EAE in wild-type mice. However, their results with nimesulid stand in contrast to investigations by Muthian et al., which demonstrated therapeutic effects with 4 different COX-2 inhibitors [36]. Other non-specific COX-2 inhibitors (indomethacin) have also been shown to have therapeutic effects in EAE [38,39]. Other enzymes involved in the generation of prostanoids have been implicated in the pathology of EAE. EAE is less severe in mice that lack the microsomal PGE synthase 1 (MPGES-1) gene that codes for the enzyme that synthesizes PGE2 from COX-derived PGH2 [40]. This finding suggests that PGE2 may be a major contributor to EAE.

Muthian et al., reported that the therapeutic effects of COX-2 inhibitors in the induction phase of EAE were due in part to immunomodulatory effects resulting from suppression of T-cell signaling through interleukin-12 (IL-12) [36]. In our studies of MS plaques, we showed that COX-2 was expressed in inflammatory macrophages and microglia in association with inducible nitric oxide synthase (iNOS) in chronic active lesions [20]. COX-2 and iNOS together, could interact to form the highly toxic peroxynitrite species which was also associated with MS plaques [41-43]. We postulated that the presence of COX-2 and iNOS in MS plaques could also contribute to the increases in local concentrations of glutamate which could lead to axonal damage and cell death of oligodendrocytes and neurons [20]. We also detected COX-2 and iNOS expression in a case of optic neuritis associated with continuing sub-clinical demyelination while on interferon therapy [22].

In the present investigation we have identified another potential mechanism by which COX-2 inhibition could impact demyelinating disease. COX-2 expression in oligodendrocytes appears to increase susceptibility to excitotoxicity in a fashion similar to that seen in neuronal excitotoxic death [17,18]. As such, expression of COX-2 in oligodendrocytes and oligodendrocyte precursor cells could have important consequences with respect to degenerative and regenerative components of MS. There may be similarities in mechanisms of excitotoxic death between neurons and oligodendrocytes. Mechanisms involving COX-2 in neuronal death have been established; however, these mechanisms for excitotoxic oligodendrocyte death remain to be elucidated. In neurons, the contribution of COX-2 to neuronal death is mediated by specific COX-2 generated prostanoids. COX catalyzes the initial reactions in the synthesis of prostanoids (PGs), prostaglandin D2 (PGD2), prostaglandin E2 (PGE2), prostaglandin F2α (PGF2α), prostacyclin (PGI2) and thromboxane (TxA2) from arachidonic acid [12]. Each of these PGs activates specific G-protein coupled receptors that, depending on the prostanoid, vary in number from one to four receptors as is seen for PGE2 (receptors EP1-4) [see [44]]. These four receptors for PGE2 (EP1-4), have distinct patterns of expression in different tissues and different pharmacological properties and each receptor is coupled to distinct intracellular signaling pathways [38].

In neuronal excitotoxic death, COX-2 generated PGE2 has been shown to be the major prostanoid responsible for the contribution of COX-2 to neuronal death *in vitro *[14] and *in vivo *[45]. Three groups have since shown that PGE2 stimulation of the EP1 prostanoid receptor is responsible for the contribution of COX-2 to NMDA-stimulated neuronal death *in vivo *[46,47] and *in vitro *[[25,48], see [49] for review]. Iadecola and colleagues further demonstrated that EP1 activation impaired the Na^+ ^- Ca^2+ ^exchanger (NCX) which helps neurons remove excess intracellular Ca^2+ ^following NMDA stimulation [46]. The resulting dysregulation of intracellular Ca^2+ ^led to overload of Ca^2+ ^in neurons and subsequent death. EP1 receptor activation has also been linked to the AKT signaling pathway that can contribute to neuronal death [50]. However, PGE2 may have opposing effects on neuronal viability depending on which receptor is activated. Activation of EP1 contributes to neuronal excitotoxic death, in contrast to activation of EP2 [51-53] and EP4 [50] which promote neuroprotection [see [49] for review].

Much less is known about how specific prostanoids and their receptors affect viability of oligodendrocytes, but similar roles may be seen for oligodendrocyte death as are seen with neurons. One study has linked specific prostanoids to viability of oligodendrocytes. The prostanoid PGD2 and its metabolite 15d-PGJ2 have been shown to directly stimulate death of oligodendrocyte precursors *in vitro *[54]. In this case, the effects of these prostanoids were independent of prostanoid receptors and linked to oxidative stress [54]. Other prostanoids (including PGE2) were tested and had no direct toxic effects on oligodendrocytes [54]. However, it is important to note that with neurons, PGE2 was necessary, but not sufficient to induce excitotoxic death. In this case, the prostanoid was not toxic by itself, but could contribute to the effect of the excitotoxin. Further investigations will be required to determine what role specific prostanoids and their receptors play in the excitotoxic death of oligodendrocytes.

Our study implicates COX-2 as a potential contributor to oligodendrocyte death and demyelination. However, the use of COX-2 inhibitors for treating MS may be complicated due to cardiovascular disease side effects associated with some COX-2 inhibitors [55,56]. An understanding of how COX-2 contributes to oligodendrocyte viability may identify new targets for treatment downstream of COX that may be safer and more effective.

## Conclusion

This study demonstrates that COX-2 expression in oligodendrocytes contributes to susceptibility to excitotoxic death. These results suggest that inhibitors of COX-2 could limit oligodendrocyte excitotoxicity and demyelination and may be considered as potential therapies for MS.

## Competing interests

The authors declare that they have no competing interests.

## Authors' contributions

NGC is the major contributor in drafting the manuscript and revising it critically for important intellectual content, conception and design, acquisition of data, analysis and interpretation of data. MAR has played a major role in revising the manuscript critically for important intellectual content conception and design, acquisition of data, analysis and interpretation of data. JWR, PT, BW and KEH played important roles in design and acquisition of data, analysis and interpretation of data. JWR has been involved in drafting the manuscript and revising it critically for important intellectual content, conception and design. All authors read and approved the final manuscript.
